# Impact of Intercropping on the Diazotrophic Community in the Soils of Continuous Cucumber Cropping Systems

**DOI:** 10.3389/fmicb.2021.630302

**Published:** 2021-03-31

**Authors:** Huan Gao, Sen Li, Fengzhi Wu

**Affiliations:** ^1^Department of Horticulture and Landscape Architecture, Northeast Agricultural University, Harbin, China; ^2^Key Laboratory of Biology and Genetic Improvement of Horticultural Crops (Northeast Region), Ministry of Agriculture and Rural Affairs, Northeast Agricultural University, Harbin, China

**Keywords:** cucumber, intercropping, diazotrophic community, *nifH*, continuous

## Abstract

Diazotrophs are important soil components that help replenish biologically available nitrogen (N) in the soil and contribute to minimizing the use of inorganic N fertilizers in agricultural ecosystems. However, there is little understanding of how diazotrophs respond to intercropping and soil physicochemical properties in cucumber continuous cropping systems. In this study, using the *nifH* gene as a marker, we have examined the impacts of seven intercropping plants on diazotrophic community diversity and composition compared to a cucumber continuous cropping system during two cropping seasons. The results showed that intercropping increased the abundance of the *nifH* gene, which was negatively correlated with available phosphorous in the fall. Diazotrophic diversity and richness were higher in the rape–cucumber system than in the monoculture. Multivariate regression tree analysis revealed that the diversity of the diazotrophic communties was shaped mainly by soil moisture and available phosphorous. *Skermanella* were the dominant genera in all of the samples, which increased significantly in the mustard–cucumber system in the fall. There was no effect of intercropping on the structure of the diazotrophic community in this case. Non-metric multidimensional scaling analysis showed that cropping season had a greater effect than intercropping on the community structure of the diazotrophs. Overall, our results suggest that intercropping altered the abundance and diversity rather than the structure of the diazotrophic community, which may potentially affect the N fixation ability of continuous cropping systems.

## Introduction

The decline of biodiversity has negative impacts on ecosystem function which related to biomass production and nutrient cycling (Cardinale et al., 2012). Modern agricultural practices trend toward crop monocultures, which results in the simplification of the components of agricultural systems (Cook, 2006; Zhou et al., 2017). The continuous monocropping system, in which the same crop is repeatedly cropped in the same field year after year, is commonly adopted in the growing and harvesting of cucumber (*Cucumis sativus* L.) (Jin et al., 2020). However, monocropping is not sustainable in the long term, because it may eventually lead to reductions in the yield and quality of the crop, a phenomenon that has been described as “soil sickness” (Yu et al., 2000).

Large amounts of chemical fertilizers have been used to increase plant biomass and grain yield in monocropping systems (Li et al., 2016). Nitrogen (N) is a critical plant nutrient, and cucumber has a very high requirement for it. Long-term continuous cropping of cucumber has been found to increase soil available nitrogen and decrease soil enzyme activities (Zhou and Wu, 2015). Application of N fertilizer promotes cucumber biomass and improves cucumber yields, however, it also incurs a significant cost to the producer and could have undesirable environmental consequences. Environmental concerns around the application of N fertilizers include increased greenhouse gas emissions, soil acidification, and groundwater contamination (Ridley et al., 2004; Hayden et al., 2010; Reardon et al., 2014). Thus, reducing N fertilizer inputs and developing new cultural practices for the improvement of agroecosystem balance is very important (Yang L. et al., 2019).

Biological nitrogen fixation (BNF) is a major source of soil nitrogen, in which atmospheric nitrogen can be reduced to ammonia by diazotrophs using nitrogenase (Yin et al., 2018). The *nifH* gene, which encodes the reductase subunit of nitrogenase, has been widely used to study diazotroph communities in terrestrial ecosystems (Hu et al., 2018; Chen et al., 2019). Soil diazotrophic community diversity and composition are sensitive to many factors related to microbial biomass (Hayden et al., 2010), soil physical and chemical properties (Poly et al., 2001; Nelson and Mele, 2006; Hayden et al., 2010; Wakelin et al., 2010), and sampling season (Mergel et al., 2001; Pereira e Silva et al., 2011). Soil diazotrophs are also sensitive to agricultural practices such as plowing, cropping systems and fertilization (Hayden et al., 2010; Berthrong et al., 2014). Although numerous factors may affect diazotrophic communities, these specific factors do not have the same effects on community diversity and composition in different cropping systems (Poly et al., 2001).

Intercropping is the growing of more than one crop species in the same field during the growing season, and it is becoming more and more important to improve soil quality and relieve soil sickness (Zhou et al., 2011; Li and Wu, 2018). Compared with monocropping, intercropping was found to increase the diversity and populations of soil bacterial communities (Li et al., 2013; Yang et al., 2016; Yu et al., 2019). The abundance and diversity of *nifH* genes showed variations in continuous and rotational soybean cropping systems (Xiao et al., 2010). Intercropping can facilitate the mobilization and uptake of N, P, and K through interspecific rhizosphere interactions (Li et al., 2003a, b; Inal et al., 2007). Several studies have indicated that intercropping can reduce nitrate leaching and the release of nitrate pollution to groundwater (Li et al., 2005; Whitmore and Schroder, 2007). However, little is known about the effects of intercropping on the diazotrophic communities in the soils of continuous cropping systems.

Previously, several vegetable species belonging to Leguminosae (Thorsted et al., 2006), Brassicaceae (Lai et al., 2019), Gramineae (Xu et al., 2013, 2015), and Compositae (Hamida et al., 2016) have been shown to have overall beneficial effects on component crop production during intercropping. Therefore, in this study, we selected seven plants from those families and intercropped with cucumber to investigate their effects on the diazotrophic community in the soils of continuous cucumber cropping systems. The diversity and composition of the diazotrophic communities were determined by Illumine MiSeq sequencing, and the abundance of diazotrophs was determined by quantitative PCR analysis of the *nifH* gene. Our previous studies showed that both cucumber growth and yield were adversely affected by continuous monocultures, while intercropping was able to both improve cucumber yield and increase the diversity of the bacterial and fungal communities (Chang et al., 2017; Li and Wu, 2018). Thus, we hypothesize that: (1) intercropping might affect the diversity and composition of the diazotrophic community; and (2) the change in the diazotrophic community are related to soil physicochemical properties.

## Materials and Methods

### Experimental Design

The experiments were conducted in a greenhouse at the Horticultural Experimental Station of the Northeast Agricultural University, Harbin, China (45°41′ N, 126° 37′ E). The field soils were subjected to continuous cropping of cucumber for 3 years. The physicochemical properties of the soil before the experiments have been previously described by Li and Wu (2018).

**FIGURE 1:**
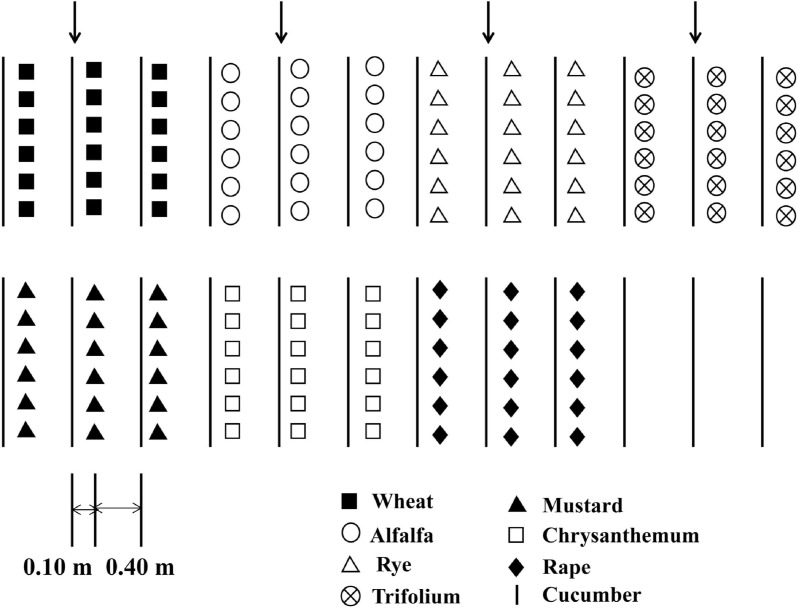
Diagram showing different intercropping systems and the cucumber monoculture system in the greenhouse experiment.

Cucumber seedlings with three true leaves were transplanted into the field, with 12 cucumber seedlings per row. The experiments were carried out in a randomized block design, with three replicate block for each treatment. There were eight treatments in the experiment: alfalfa (A), trifolium (T), wheat (W), rye (Ry), chrysanthemum (C), rape (Ra), and mustard (M) were intercropped with cucumber as an experimental group, and cucumber monoculture (CM) as the control. First cropping of cucumber was grown in 2015. Spring and fall experiments were conducted from April to June and from July to October, respectively. Twelve cucumber were planted in one row as the test district. There were two protective rows on both sides. Ten days following the planting of the cucumber, the intercrop seeds were sown on the outside of the rows of cucumber at a distance of approximately 0.10 m. The distance between the two rows cucumber was 0.50 m (Figure 1).

Each cucumber seedling was intercropped with (I) 30 wheat and rye seedlings, respectively, (II) 40 trifolium and alfalfa seedlings, respectively, (III) 3 rape and mustard seedlings, respectively, (IV) 5 chrysanthemum seedlings. The seedlings were irrigated with groundwater twice a week. NPK compound fertilizer (16:16:8) was applied with 300 kg ha^–1^ in each plot. The weeds were removed manually and no pesticide were applied. A more detailed description of the experiment is available in the references cited (Chang et al., 2017; Li and Wu, 2018).

### Soil Sampling, Microbial Biomass, and Soil Enzyme Activities Analysis

A total of 48 soil samples were collected from 24 test districts on June 30 and October 6. The soil microbial biomass C and N were measured by using the chloroform fumigation-extraction method (Brookes et al., 1985; Vance et al., 1987). The soil urease activity was measured according to Zhou et al. (2011). The soil protease activity was measured as described by Ladd et al. (1976).

### DNA Extraction and *nifH* Gene Copy Number Quantification

Soil DNA was extracted from 0.5 g soil using a powerful DNA Isolation Kit (Mo Bio Laboratories Inc., Carlsbad, CA, United States) according to the manufacturer’s instructions. DNA quality was assessed using 1% agarose gel electrophoresis. *nifH* gene copy numbers were determined using qPCR assays in an IQ5 real-time PCR system (Bio-Rad Laboratories, Inc., Hercules, CA, United States) with Po1F/Po1R (Poly et al., 2001). The PCR reaction of diazotroph including 10 μl of 2 × Real SYBR Mixture (Tiangen Beijing, China), 0.8 μl of 10 μM forward and reverse primers, 7.2 μl of sterilized MilliQ water, and 2 μl of DNA template. A negative control reactions contained 2 μl of H_2_O instead of DNA. Amplification was initiated by denaturation at 95°C for 3 min, followed by 45 cycles of denaturation at 95°C for 15 s, annealing at 55°C for 45 s, extension at 72°C for 30 s, and a final elongation at 72°C for 10 min, and the plate was read at 83°C. The specificity of the amplicon was verified by melting curve analysis and agarose gel electrophoresis.

### Illumina Miseq Sequencing and Bioinformatics Analysis

The *nifH* gene was amplified with the primer pairs *nifH*-F/*nifH*-R (Rösch et al., 2002). The reaction composition and thermal conditions of the PCR were conducted according to the method of Fan et al. (2019). The PCR products obtained were purified with an Agarose Gel DNA purification kit (TaKaRa Bio), and triplicate PCR amplifications for each sample were conducted and pooled as a PCR product and then sequenced on an Illumina Miseq platform at Majorbio Bio-Pharm Technology Co., Ltd. (Shanghai, China). The raw sequences were processed using the QIIME-1.9.1 pipeline (Edgar, 2013). The low-quality sequences were removed, as described previously by Fan et al. (2019), and the remaining sequences were further translated into amino acids using the FunGene pipeline. The translated protein and chimera sequences that did not match the nifH protein sequence were discarded. The remaining high-quality sequences were clustered into operational taxonomic units (OTUs) at 95% similarity using Uparse software (version 7.0.1090). The raw data were uploaded to the NCBI SRA database with the submission accession number SRP159857. The read numbers of all the samples were normalized to the same sequencing depth, and the Chao1, Observed species, and Shannon and Simpson indices were chosen to evaluate alpha diversity (Yang Y. et al., 2019).

### Data Analysis

Mean comparison (microbial biomass C and N, soil enzyme activities, *nifH* gene abundance, number of OTUs, alpha-diversity) of the different treatments was performed based on the Tukey’s honest significance difference (HSD) test at the 0.05 probability level. The *nifH* gene abundance was analyzed using two-way analysis of variance (ANOVA) with season, cropping system, and their interaction as the fixed factors. These statistical analyses were conducted with SAS 9.2 software (SAS Institute Inc., Cary, NC, United States). Spearman correlation coefficients were calculated in order to test the relationships between the soil physicochemical properties, the *nifH* gene abundance, and the relative abundance of genera in SPSS software (Version 17.0). Non metric multidimensional scaling (NMDS) and redundancy analysis (RDA) were conducted to reveal the structure of the diazotroph community and the relationship between soil environmental factors and soil diazotroph community abundances. Analysis of similarity (Anosim) and non-parametric MANOVA (Adonis) were used to compare the differences in the microbial communities of the two cropping seasons using the Bray–Curtis distance and 999 permutations, and were carried out in R using the vegan package. A multivariate regression tree analysis was performed to identify the most important soil factors for diazotroph diversity using R-package “mvpart.”

## Results

### Microbial Biomass and Soil Enzyme Activities

Compared with the monoculture system, the wheat–cucumber system in the two growing seasons, and rye-cucumber and chrysanthemum–cucmber systems in the fall significantly increased microbial biomass C. The microbial biomass C of alfalfa–cucumber and chrysanthemum–cucmber systems was significantly lower than those of the monoculture in the spring. The chrysanthemum–cucmber and mustard–cucumber systems significantly increased the microbial biomass N in the two growing seasons compared with the monoculture. The microbial biomass N of alfalfa–cucumber, trifolium–cucumber, wheat-cucumber, and rape–cucumber systems was significantly lower than those of the monoculture in the fall. Compared with the monoculture system, rape–cucumber system significantly increased the urease activity in the fall and all intercropping systems significantly increased the protease activity in the two growing seasons (Supplementary Table 1).

### Abundance of the *nifH* Gene and Its Correlation With the Soil Properties

**FIGURE 2:**
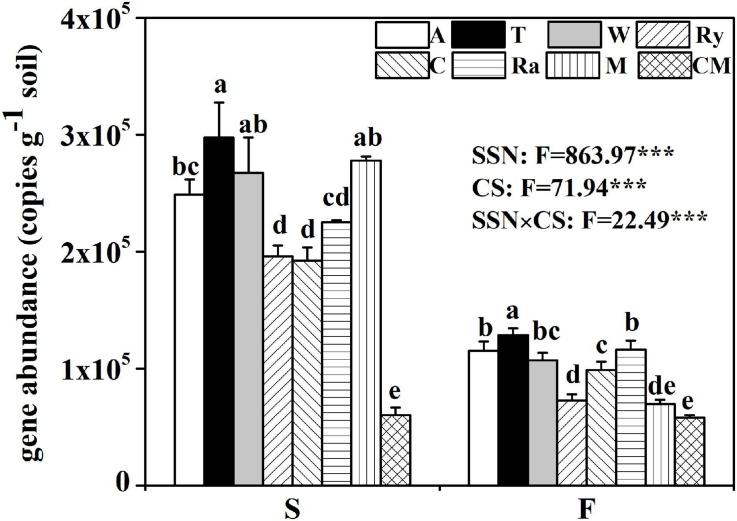
Abundance of the *nifH* gene among eight cropping systems in the spring (S) and fall (F) cropping seasons. Data with different letters in each column indicate significant differences among the samples (*p* < 0.05, one-way ANOVA). *F-*value from two way ANOVA analysis of effects of season and cropping system on *nifH* gene abundance. SSN, season; CS, cropping system.

Compared with the cucumber monoculture, the *nifH* gene abundance significantly increased under the intercropping systems in the two growing seasons except for the mustard–cucumber system in the fall. Moreover, the trifolium–cucumber system had the highest *nifH* gene abundance in the two growing seasons. Two-way analysis showed that season, cropping system and their interaction had a significant effect on *nifH* gene abundance (Figure 2). No significant correlation was observed between *nifH* gene abundance and soil properties measured in the spring. In the fall, a significant negative correlation was observed between *nifH* gene abundance and available phosphorous (AP). Regardless of the season, *nifH* gene abundance was positively correlated with pH and nitrate (Table 1).

**TABLE 1:** Correlation coefficients for relationships between *nifH* gene abundance and soil physicochemical properties.

***nifH* gene**	**pH**	**NH_4_^+^**	**NO_3_^–^**	**AP**	**AK**	**Moisture**	**EC**	**MBC**	**MBN**
Spring	0.243	–0.199	0.101	–0.281	–0.281	–0.236	–0.003	0.200	0.133
Fall	0.134	–0.070	0.188	−0.408*	–0.083	0.306	–0.105	–0.104	–0.244
Overall	0.721**	0.184	0.295*	–0.129	0.093	–0.049	–0.017	0.105	–0.240

AP, soil available phosphorus; AK, soil available potassium; MBC, microbial biomass carbon; MBN, microbial biomass nitrogen; “Overall,” regardless of season (*p < 0.05; **p < 0.01).

### Diazotrophic Community Diversity and Composition

**FIGURE 3:**
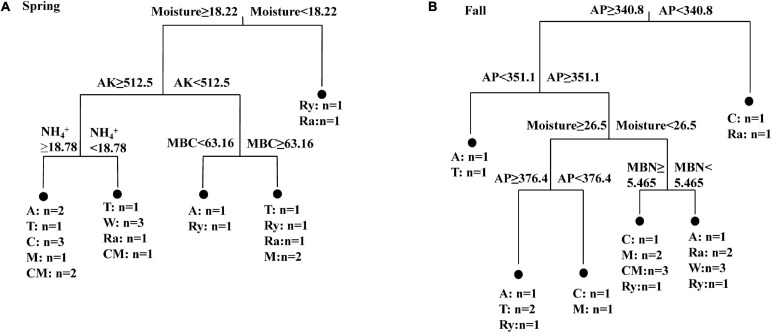
Multivariate regression tree analysis of alpha diversity (OTUs, Chao 1, Shannon and Simpson indices) of diazotrophs and soil physicochemical properties in the **(A)** Spring and **(B)** Fall cropping seasons. Treatments and the number of samples included in the analysis are shown at the bottom. AK, available potassium; AP, available phosphorus; MBC, microbial biomass carbon; MBN, microbial biomass nitrogen.

The alpha-diversity indices of the diazotrophic community are listed in Table 2. The number of OTUs in the rape–cucumber system was significantly higher than that in the monoculture in either the spring or the fall. Compared with the monoculture system, the mustard–cucumber system in the spring and the wheat–cucumber and chrysanthemum–cucumber systems in the fall had significantly increased the numbers of OTUs. The Shannon indices of the wheat–cucumber, rape–cucumber, and mustard–cucumber systems were significantly higher than those of the monoculture in both the spring and the fall. However, the Simpson index showed the opposite trend to the Shannon index. The Chao1 index was used to estimate the diazotrophic richness. The Chao1 index of the rape-cucumber system was significantly higher than those of the monoculture in the spring and the fall. The coverage of the diazotrophic community was more than 96%, showing that the sequencing depth in this report was enough to cover the soil diazotrophic community (Table 2). Two-way analysis showed that both the number of OTUs and the alpha-diversity of the diazotrophic community were affected mainly by the cropping system and the interaction between season and cropping system (Table 2). Multivariate regression tree analysis indicated that diazotrophic diversity and richness were mainly shaped by soil moisture in the spring and available phosphorus in the fall (Figure 3).

**TABLE 2:** Number of OTUs and diversity indices of the diazotrophic communities in the soils of cucumber crops for all treatments.

**Crop season**	**Treatments**	**Number of OTUs**	**Shannon**	**Simpson**	**Chao1**	**Coverage (%)**
S	A	607 ± 49 c	3.94 ± 0.14 e	0.057 ± 0.011 a	1,047 ± 26 bc	96.60
	T	707 ± 49 bc	4.36 ± 0.06 bc	0.034 ± 0.002 cd	1,199 ± 131 abc	96.60
	W	661 ± 1 bc	4.25 ± 0.04 bc	0.034 ± 0.004 cd	1,215 ± 109 ab	96.84
	Ry	719 ± 35 bc	4.23 ± 0.17 cd	0.042 ± 0.009 bc	1,296 ± 64 a	96.49
	C	636 ± 7 c	4.22 ± 0.00 cd	0.036 ± 0.000 bcd	982 ± 23 c	97.12
	Ra	858 ± 23 a	4.80 ± 0.12 a	0.024 ± 0.003 d	1,362 ± 200 a	97.02
	M	768 ± 34 ab	4.44 ± 0.08 b	0.030 ± 0.004 cd	1,270 ± 99 ab	96.94
	CM	612 ± 59 c	4.03 ± 0.07 de	0.050 ± 0.013 ab	1,047 ± 87 bc	96.70
F	A	658 ± 89 bc	3.92 ± 0.37 cd	0.069 ± 0.025 ab	1,134 ± 121 c	96.45
	T	591 ± 10 c	3.59 ± 0.01 d	0.099 ± 0.004 a	923 ± 68 d	96.96
	W	848 ± 16 a	4.72 ± 0.24 ab	0.031 ± 0.009 c	1,242 ± 28 bc	96.90
	Ry	746 ± 57 b	4.30 ± 0.32 bc	0.051 ± 0.022 bc	1,182 ± 50 c	96.35
	C	891 ± 50 a	4.97 ± 0.20 a	0.019 ± 0.002 c	1,333 ± 45 b	96.66
	Ra	876 ± 2 a	4.52 ± 0.16 ab	0.049 ± 0.020 bc	1,460 ± 58 a	96.27
	M	717 ± 27 b	4.38 ± 0.04 b	0.032 ± 0.002 c	1,246 ± 67 bc	96.88
	CM	691 ± 25 b	3.86 ± 0.22 cd	0.081 ± 0.017 ab	1,191 ± 15 c	96.63
Seasons (SSN)		17.37***	0.00 ns	16.51***	1.58 ns	
Cropping system (CS)		16.79***	14.07***	9.13***	7.53***	
SSN × CS		9.91***	8.37***	6.12***	5.05***	

Data with different letters in each column represent significant differences between treatments at the 0.05 level. F-values from two-way ANOVA analysis of effects of season and cropping system on the number of OTUs and diversity indices of the diazotrophic communities. ***p < 0.001, ns, not significant.

**FIGURE 4:**
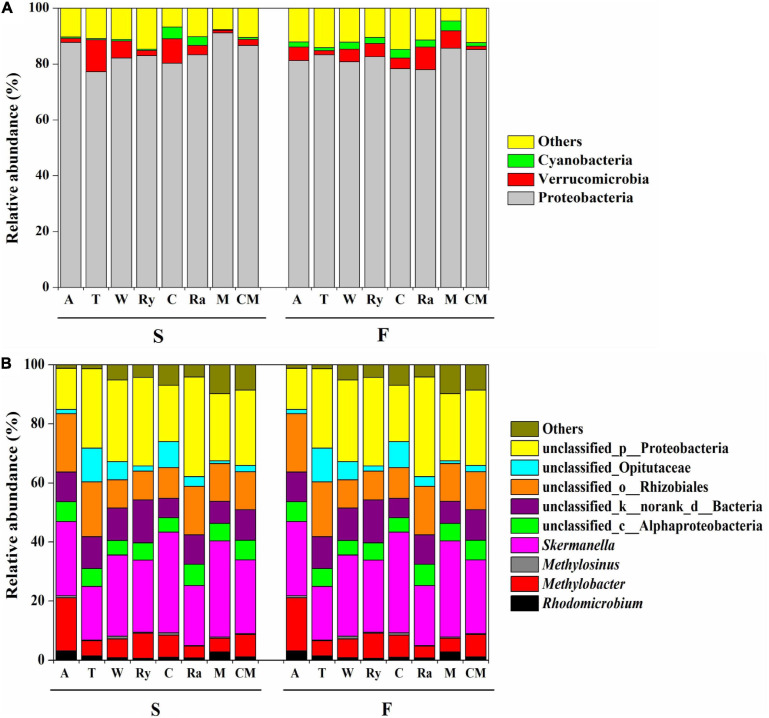
Relative abundances (%) of **(A)** diazotrophic phyla and **(B)** genera among eight cropping systems in the spring (S) and fall (F) cropping seasons. “Others” includes phyla and genera below 0.5% relative abundance.

All of the samples were dominated to a great degree by the phylum Proteobacteria, with relative abundances ranging from 67.11 to 95.50%, followed by Verrucomicrobia (11.39–0.94%), and Cyanobacteria (4.17–0.24%). Compared with the monoculture, the relative abundances of these phyla showed no significant changes in response to the intercropping system except for the Cyanobacteria in the chrysanthemum–cucumber system in the spring (Figure 4A).

Figure 4B shows the relative abundances of the four main soil diazotrophic genera in the two growing seasons. We found that different intercropping regimes resulted in different changes in the diazotrophic communities at the genus level in the two growing seasons. For example, the rye–cucumber system significantly increased the relative abundance of *Rhodomicrobium* in the fall, and the alfalfa–cucumber system significantly increased the relative abundances of *Methylobacter* and *Methylosinus* in spring compared with the monoculture. The mustard–cucumber system significantly increased the relative abundance of *Skermanella* in the fall compared with the monoculture (Supplementary Figure 1).

**FIGURE 5:**
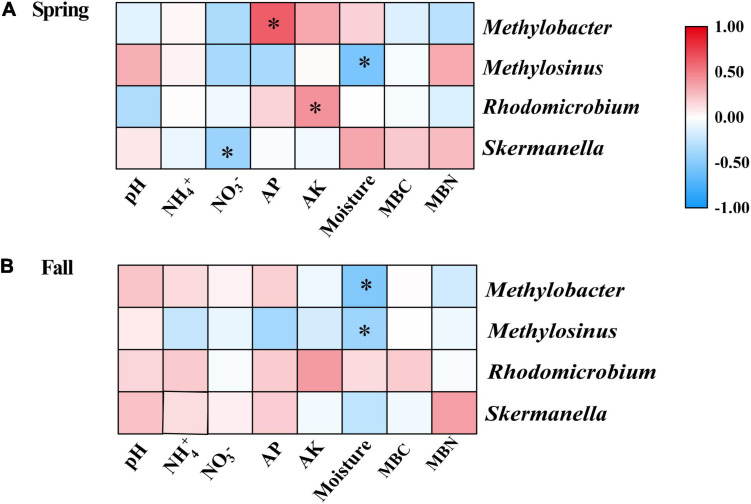
Spearman correlation analysis of the relative abundances of dominant diazotrophic genera and soil physicochemical variables in the **(A)** Spring and **(B)** Fall cropping seasons. *p < 0.05. AK, available potassium; AP, available phosphorus; MBC, microbial biomass carbon; MBN, microbial biomass nitrogen.

The results of the Spearman correlation analysis showed that several properties had significant relationships with the relative abundance of some diazotrophic genera in individual seasons (Figures 5A,B). In the spring, the relative abundance of *Methylobacter* and *Rhodomicrobium* were significantly positively correlated with AP and AK, respectively; the relative abundance of *Methylosinus* and *Skermanella* were significantly negatively correlated with moisture and nitrate, respectively (Figure 5A). In the fall, the relative abundances of *Methylobacter* and *Methylosinus* were significantly negatively correlated with moisture (Figure 5B).

### Structure of the Diazotrophic Community and Its Correlation With Soil Properties

**FIGURE 6:**
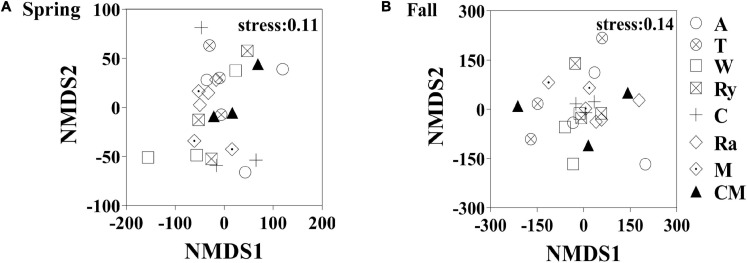
Non-metric multidimensional scaling (NMDS) analysis of the diazotrophic communities in the **(A)** spring (S) and **(B)** Fall (F) cropping seasons.

**FIGURE 7:**
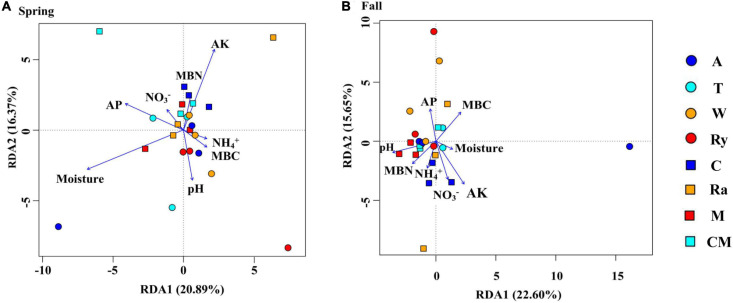
Redundancy analysis (RDA) showing correlations between soil diazotrophic communities and soil environmental factors during the **(A)** Spring and **(B)** Fall cropping seasons in the control (CM), alfalfa (A), trifolium (T), wheat (W), chrysanthemum (C), rye (Ry), mustard (M), and rape (Ra) intercropping systems.

Non-metric multidimensional scaling (NMDS) analysis based on the abundance of OTUs revealed differences in the structure of the diazotrophic community (Figures 6A,B). The NMDS plot based on the Euclidean distance dissimilarity showed that the three replicates per treatment were not situated close together, and the soil diazotrophic communities of all samples were distinctly different in the two growing seasons (Table 3). Moreover, there was no obvious distinction between the intercropped systems and the monoculture in the fall (Figure 6B), but the rape–cucumber system was separated from the monoculture in the spring (Figure 6A). Redundancy analysis (RDA) (Figure 7) and the Monte Carlo permutation test (Table 4) were carried out in order to examine the relationship between environmental factors and the soil diazotrophic community composition. Among the environmental factors, AK was significantly correlated with the soil diazotrophic community composition in the two growing seasons (Table 4). Moisture and AP were significantly correlated with the composition of the soil diazotrophic community in the spring and pH, NO_3_^–^, and MBC were significantly correlated with the soil diazotrophic community composition in the fall. However, NH_4_^+^ and MBN were not significantly correlated with soil diazotrophic community composition (Table 4).

**TABLE 3:** Dissimilarity comparison of the soil diazotrophic community structure between the spring and fall cropping seasons.

**Spring vs. Fall**	**Anosim**		**Adonis**		
	***R***	***p***	**F**	***R*^2^**	***p***
Diazotrophic community	0.423	0.001	8.44	0.235	0.001

**TABLE 4:** Monte Carlo permutation test of environmental factors and soil diazotrophic community composition.

**Variable**	**S**				**F**			
	**RDA1**	**RDA2**	***r*^2^**	***p***	**RDA1**	**RDA2**	***r*^2^**	***p***
pH	0.19858	–0.98009	0.1738	0.143	–0.96424	–0.26501	0.3288	**0.007^∗∗^**
NH_4_^+^	0.94892	–0.31553	0.0455	0.546	–0.26350	–0.96466	0.1457	0.166
NO_3_^–^	–0.66596	0.74599	0.0494	0.570	0.28287	–0.95916	0.3008	**0.025^∗^**
AP	–0.92332	0.38402	0.3005	**0.016^∗^**	–0.17010	0.98543	0.2029	0.082
AK	0.38768	0.92179	0.5088	**0.002^∗∗^**	0.51124	–0.85944	0.4814	**0.001^∗∗∗^**
Moisture	–0.93916	–0.34349	0.7875	**0.001^∗∗∗^**	0.87923	–0.47640	0.0586	0.387
MBC	0.83155	–0.55545	0.0784	0.403	0.60374	0.79718	0.2566	**0.036^∗^**
MBN	0.21930	0.97566	0.0915	0.348	–0.67954	–0.73364	0.1849	0.088

Only significant correlations (*p < 0.05; **p < 0.01; ***p < 0.001) are shown in bold. AP, available phosphorus; AK, available potassium; MBC, microbial biomass carbon; MBN, microbial biomass nitrogen.

## Discussion

### Effect of Intercropping on Soil Diazotrophic Abundance

Intercropping is a sustainable farming model that have an effect on crop growth (Hauggaard-Nielsen and Jensen, 2005), microbial communities (Li et al., 2016), and microclimatic conditions (Cong et al., 2015). BNF is a major source of soil nitrogen, in which atmospheric nitrogen can be reduced to ammonia by diazotrophs using nitrogenase (Yin et al., 2018). In this study, we found that intercropping can lead to an increase in *nifH* gene abundance compared with monoculture systems (Figure 2). Previous study also found that intercropping can promote the population of bacteria associated with nitrogen fixing (Chen et al., 2018). Enhanced diazotrophic abundance in the present study may be caused by interspecific facilitations in the intercropping systems. It has been reported that plant species could alter the soil bacterial communities (Rooney and Clipson, 2008; Junier et al., 2009). Our observation that the trifolium–cucumber system had the highest *nifH* gene abundance in the two growing seasons (Figure 2). Normally, nitrogen fixation rate is correlated with *nifH* gene abundance (Reed et al., 2010), and it is possible that the higher *nifH* gene abundance, due to the presence of symbiotic N-fixers in cucumber intercropped with leguminous crops (Yang Y. et al., 2019), may improve the nitrogen fixation in such treated soil. However, Rocca et al. (2015) found that the higher abundance of the gene did not always lead to higher metabolic rates in soils. Therefore, the BNF rate of intercropping systems needs to be determined in future studies.

Diazotrophic community abundance is sensitive to the soil physicochemical characteristics (Hayden et al., 2010). In our study, a significant negative correlation was observed between *nifH* gene abundance and AP in the fall (Table 1). Phosphorus is one of the crucial factors controlling nitrogen fixation due to the high demand for adenosine triphosphate in BNF (Hill, 1988; Vitousek et al., 2002; Reed et al., 2011). A previous study has shown that soil nitrogenase activity was positively correlated with soil total phosphorus (Han et al., 2019). Thus, we suggest that the lower *nifH* gene abundance of rye–cucumber, mustard–cucumber, and monoculture systems in the fall may be attributed to the higher AP, while the nitrogenase activity in these treatments may higher than in others. Regardless of season, pH was positively significantly correlated with the *nifH* gene abundance (Table 1). Soil pH is a key factor affecting *nifH* gene abundance, and a high pH value favors BNF (Nelson and Mele, 2006). Thus, we deduced that the higher abundance of the *nifH* gene observed in the spring than in the fall was ascribed to the higher soil pH in the spring. A similar result was reported by Pereira e Silva et al. (2013), who found that pH was positively correlated with *nifH* gene abundance in soils with pH values ranging from 4.3 to 7.7. The change of pH may influence the bioavailability of carbon or nitrogen sources in soil which may be one possible reason why pH value affect the growth of diazotrophs (Lauber et al., 2009). N availability may have a negative impact on diazotroph abundance (Reardon et al., 2014; Zhalnina et al., 2015). In our study, however, the NH_4_^+^ availability was not different between the intercropping and monoculture except for the rye-cucumber system in the spring (Li and Wu, 2018). The increase in soil protease activities in all intercropping systems were not accompanied by increased in soil NH_4_^+^ availability, probably due to the plant uptake or N losses. Moreover, our study found that *nifH* gene abundance was positively correlated with NO_3_^–^ regardless of the season (Table 1). This might be due to the fact that soils with high total nitrogen may be helpful for sustaining better plant growth and thus supply more plant carbon substrates to nourish diazotrophs, as the latter are highly dependent on the availability of carbon (Han et al., 2019).

It has been shown that temporal variation can affect the diazotrophic abundance (Pereira e Silva et al., 2011, 2013). Consistently, we observed significant seasonal changes of the *nifH* gene abundance in the intercropping systems (Figure 2). Temperature may induce the variation of energy influx in different seasons due to its effect on soil bacterial communities (Pettersson and Bååth, 2003; Pereira e Silva et al., 2013). BNF is an energy consuming process (Pereira e Silva et al., 2013), it may trend to increase with increasing temperature and energy input, supporting the higher *nifH* gene abundance in the spring. All these results indicate that the abundance of diazotroph is not affected by any single factor, but by several correlated factors.

### Effect of Intercropping on Diazotrophic Communities

Intercropping could increase plant diversity, different plant species usually secrete different kinds and amounts of root exudates, which can exert specific effects on soil microbial community diversity and composition (Grayston et al., 1998; Arafat et al., 2017; Chen et al., 2018; Li and Wu, 2018; Rahman et al., 2020). We observed that the alpha-diversities of diazotrophic communities in the rape–cucumber and mustard–cucumber systems were significantly higher than that of the monoculture (Table 2). Rape and mustard both belong to the Brassicaceae, and they could release glucosinolates along with their degradation products through root exudation, which can directly impact soil microbes (Choesin and Boerner, 1991; Schreiner and Koide, 1993; Hanschen et al., 2015; Jin et al., 2019). A previous study has shown that glucosinolates alter bacterial communities through an increased relative abundance of bacteria able to use these compounds as an additional C-source (Hanschen et al., 2015). Thus, our results imply that specific compounds in the Brassicaceae may affect diazotrophic community diversity. Soil microbial diversity is closely related to nutrient cycling (Giuditta et al., 2019). In our study, we found that the activities of N cycle related enzymes (urease and protease) in rape–cucumber system were highest in the fall (Supplementary Table 1), while the increase in activities of urease and protease did not cause an increase in available NH_4_^+^ (Li and Wu, 2018). We suggest this might be due to the high plant uptake, which needs to be investigated in further studies.

Multivariate regression tree analysis showed that diazotrophic diversity and richness were mainly shaped by soil moisture in the spring (Figure 3A). This result was in line with a previous finding reported by Che et al. (2018). Water content can affect soil microbial communities directly or indirectly by affecting the availability and quality of organic carbon (Che et al., 2018). Soil P nutrient is one of the important factors in regulating the change of diazotrophic diversity and richness (Yang L. et al., 2019). Similar result was found in our present study where the diazotrophic diversity in the fall was mainly changed by soil AP content (Figure 3B). Taken together, our results suggest that both the soil moisture and AP are important factors in regulating the changes of the diazotrophic diversity and richness.

The *nifH* community was dominated by Proteobacteria in terms of relative abundance, followed by Verrucomicrobia and Cyanobacteria, whereas the presence of other phyla were found to be negligible in our study (Figure 4A). This finding is in line with those of a previous study, namely, that Proteobacteria were one of the most commonly diazotrophs found in terrestrial ecosystems (Zehr et al., 2003). In addition, a study by Quesada et al. (1997) showed that Cyanobacteria play key roles in BNF in rice-field soils. However, the abundance of soil bacterial at the phylum level does not always reveal the functional features of sites, and, consequently, further analysis we focus on the genus level.

In this study, we discovered that *Skermanella* were the most abundant diazotrophic genera in all of the soil samples tested (Figure 4B). *Skermanella* belong to the Proteobacteria phylum, which are obligate chemo-organotrophs and facultative anaerobes, unable to fix nitrogen (Zhu, 2014). We found that the relative abundances of *Skermanella* in the mustard–cucumber system were slightly increased in spring and significantly increased in the fall compared with the monoculture (Supplementary Figure S1). This may have been due to the lower level of soil NO_3_^–^ in the mustard–cucumber system, since the relative abundance of *Skermanella* was negatively correlated with NO_3_^–^ concentration (Figure 5A). Moreover, a previous study has shown that the presence of *Skermanella* was correlated with the level of the phenolic-related compound gamma-aminobutyric acid GABA from Arabidopsis root exudates (Badri et al., 2013). Arabidopsis and mustard both belong to the Brassicaceae, and this component may also be present in the root exudates of mustard, which affects the abundance of *Skermanella*.

*Methylobacter* and *Methylosinus* belong to Type I and Type II methanotrophs, respectively (Zhang et al., 2014). Both methanotrophs possess nitrogen fixing genes, and both are able to fix N_2_ under laboratory experimental conditions (Auman et al., 2001; Boulygina et al., 2002; Dedysh et al., 2004). The significant correlations between soil moisture and the abundance of *Methylobacter* in the fall (Figure 5B) and of *Methylosinus* in both growing seasons (Figures 5A,B) implied that these two diazotrophic genera were very sensitive to soil moisture. The alfalfa–cucumber system significantly increased the relative abundances of *Methylobacter* and *Methylosinus* in spring compared with the monoculture (Supplementary Figure 1). Specific flavonoids in the root exudates of leguminous crops, which act as signaling molecules to attract N-fixing bacteria, might be the reason for the diazotrophic abundance changes in alfalfa–cucumber system (Broughton et al., 2003). *Rhodomicrobium* as photosynthetic bacteria, have been shown to be capable of fixing N (Lindstrom et al., 1950). The significant correlations between the relative abundance of *Rhodomicrobium* and soil AK (Figure 5A) imply that this diazotrophic genus is sensitive to the soil concentration of potassium nutrients.

The *Bradyrhizobium* genus is ubiquitous in soil, and includes symbiotic N-fixing bacterial species and free-living soil diazotrophs. The relative abundance of *Bradyrhizobium* was found to be lower than 0.5% in our study (data not shown), and this lower relative abundance may be attributed to the higher pH of the soil in our study. Previous studies have reported that indigenous *Bradyrhizobium* abundance decreased with an increase in soil pH, especially when the soil pH was greater than 6.0 (Tang and Robson, 1993; Rossum et al., 1994; Zhalnina et al., 2013). Yet, when soil nitrogen was not limiting to the plant, this symbiosis did not occur (Omrane and Chiurazzi, 2009). Compared with the result of the study by Lin et al. (2018), the soil nitrogen content found in our study was higher. This may have been the reason why the genera measured in our study were non-symbiotic diazotrophs. Further research is required to evaluate their N-fixing capacity.

NMDS and RDA showed that the structure of the soil diazotrophic community did not change significantly between intercropped and monocultured soils (Figures 6, 7). However, the diazotrophic community structure changed significantly in the two growing seasons of spring and fall (Table 3). The pH of the soil did not differ significantly among the treatments, but increased in the fall compared with the spring. This suggests that pH was the predominant factor driving seasonal changes in the diazotroph community. Moreover, previous studies have shown that temperature affected the structure of soil diazotrophic communities (Wang et al., 2015; Lin et al., 2018). In northeastern China, significant differences in temperature exist between the two growing seasons. Therefore, we also speculate that the variation in diazotrophic community structure in the two growing seasons is related to temperature. Poly et al.’s (2001) study showed that the composition of N-fixers is influenced by the soil physicochemical characteristics. In our study, however, the soil pH, AK, and moisture, which primarily related to the diazotrophic communities were identical between the intercropped and monocultured systems. Thus, the finding that the structure of the soil diazotrophic community was not significantly affected by intercropping may have been a result of the lack of an effect of intercropping on the soil physicochemical characteristics in continuous cropping soil. Further experiments need to be carried out over the longer term in order to confirm these results.

## Conclusion

In summary, the results of our study have shown that intercropping increases *nifH* gene abundance. The rape–cucumber system significantly increased diazotrophic diversity and richness. However, intercropping had no effect on diazotrophic community structure. Moreover, we found that changes in the soil diazotrophic community were related to environmental factors, indicating that the effect of intercropping on the soil diazotrophic community was indirectly affected by the physicochemical properties of the soil. Changes in abundance and diversity rather than the structure of the diazotrophic community in intercropping systems might potentially affect the N-fixing ability of continuous cropping systems, and this hypothesis needs to be explored further.

## Data Availability Statement

The datasets presented in this study can be found in online repositories. The names of the repository/repositories and accession number(s) can be found below: https://www.ncbi.nlm.nih.gov/, SRP159857.

## Author Contributions

FW contributed to design this experiment. HG and SL performed the experiment. HG analyzed the data and wrote the manuscript. All authors have read and approved the submitted version.

## Conflict of Interest

The authors declare that the research was conducted in the absence of any commercial or financial relationships that could be construed as a potential conflict of interest.

## Supplementary Material

Supplementary Figure 1 Relative abundance (%) of the domiant genera among eight cropping systems in spring (S) and fall (F) cropping seasons.

Supplementary Table 1 Soil physicochemical properties and soil enzymes among eight cropping systems in spring (S) and fall (F) cropping seasons.
