# Supplementary material for: Impact of Intercropping on the Diazotrophic Community in the Soils of Continuous Cucumber Cropping Systems
Source: Front Microbiol. 2021 Mar 31;12:630302. doi: 10.3389/fmicb.2021.630302 (PMC8044418; doi:10.3389/fmicb.2021.630302)
Supplement: Supplementary Figure 1 — Relative abundance (%) of the domiant genera among eight cropping systems in spring (S) and fall (F) cropping seasons. [file Table_1.docx]

Table S1 Soil physicochemical properties and soil enzymes among eight cropping systems in spring (S) and fall (F) cropping seasons

| Crop season^a^ | Treatment^b^ | Microbial biomass C  （mg/kg） | Microbial biomass N  （mg/kg） | Urease  (NH_4_^+^-N mg g^-1^ soil 24 h^-1^ ) | Protease  (mg tyr g^-1^ h^-1^) |
| --- | --- | --- | --- | --- | --- |
| S | A | 47.37±15.73 d | 1.79±0.78 c | 8.08±0.36 a | 74.38±1.96 a |
|  | T | 110.53±15.69 b | 2.70±0.88 c | 6.95±1.09 a | 27.01±1.86 f |
|  | W | 142.11±15.80 a | 4.64±0.13 b | 7.86±0.58 a | 33.63±2.50 e |
|  | Ry | 102.63±7.79 bc | 2.33±1.19 c | 7.38±0.94 a | 45.45±2.88 d |
|  | C | 39.47±7.89 d | 12.73±0.97 a | 7.95±0.24 a | 49.49±5.73 cd |
|  | Ra | 78.95±15.89 c | 2.67±0.78 c | 7.77±0.26 a | 52.04±1.08 c |
|  | M | 89.47±9.12 bc | 4.89±1.63 b | 7.59±1.14 a | 65.76±4.53 b |
|  | CM | 94.74±27.30 bc | 1.95±0.56 c | 7.02±0.69 a | 18.31±1.00 g |
|  |  |  |  |  |  |
| F | A | 47.46±15.29 b | 2.53±0.13 d | 5.41±0.16 ab | 27.25±1.14 e |
|  | T | 47.37±27.35 b | 5.11±0.11 c | 5.22±0.43 ab | 27.56±1.37 e |
|  | W | 118.42±7.89 a | 2.60±1.10 d | 4.22±1.67 ab | 34.91±0.30 d |
|  | Ry | 92.11±12.06 a | 5.71±0.61 bc | 5.38±0.76 ab | 26.47±2.80 e |
|  | C | 110.63±15.39 a | 8.81±0.77 a | 5.04±0.23 ab | 50.30±2.69 b |
|  | Ra | 47.47±15.79 b | 5.22±0.55 c | 6.14±0.86 a | 66.30±3.98 a |
|  | M | 40.47±7.98 b | 8.84±0.26 a | 5.12±0.64 ab | 43.39±2.20 c |
|  | CM | 55.26±7.89 b | 6.61±0.07 b | 4.17±0.16 b | 17.16±1.89 f |

^a^S and F indicated experiment conducted in spring and fall seasons, respectively.
^b^A, Alfalfa; T, Trifolium; W, Wheat; Ry, Rye; C, Chrysanthemum; Ra, Rape; M, Mustard; CM, Cucumber monoculture.

Data with different letters in each column indicate significantly different between treatments at 0.05 level.
